# QTL mapping using an ultra-high-density SNP map reveals a major locus for grain yield in an elite rice restorer R998

**DOI:** 10.1038/s41598-017-10666-7

**Published:** 2017-09-07

**Authors:** Manshan Zhu, Dilin Liu, Wuge Liu, Dan Li, Yilong Liao, Jinhua Li, Chongyun Fu, Fuhong Fu, Huijun Huang, Xueqin Zeng, Xiaozhi Ma, Feng Wang

**Affiliations:** 1 Rice Research Institute, Guangdong Academy of Agricultural Sciences, Guangzhou, 510640 China; 2 Guangdong Key Laboratory of New Technology in Rice Breeding, Guangzhou, 510640 China

## Abstract

To dissect the genetic basis of yield formation in restorer line of hybrid rice, we conducted QTL analysis for 6 yield traits including panicles per plant (PPP), grains per panicle (GPP), grain yield per plant (GY), thousand-grain weight (TGW), above-ground biomass (AGB), and harvest index (HI) using SNP markers in a recombinant inbred lines (RILs) population derived from a cross between a tropical *japonica* inbred Francis and an elite *indica* restorer Guanghui 998 (R998). A total of 26 QTLs were detected using a high density genetic map consisting of 3016 bin markers. Nineteen out of the 26 QTL alleles from R998 had a beneficial effect on yield traits. Most of the QTLs were co-located with previously reported rice QTLs. *qAGB6* and *qHI9*, controlling AGB and HI respectively, were detected as novel QTLs. Four QTLs for GY were repeatedly detected across two years, with all the beneficial alleles from R998. Notably, *qGY8* explained over 20% of the yield variance in both years. Moreover, *qGY8* together with *qTGW8* and *qHI8* formed a QTL cluster. Markers tightly linked with *qGY8* were developed. Cloning of *qGY8* will facilitate its further exploitation in high-yield breeding.

## Introduction

Rice is a staple food crop feeding over half of the population in the world. With the increase of population and decrease of arable land, food production is facing huge challenge over the next 20 years. Hence, developing high-yielding new varieties remains one of the major goals in rice breeding. In the last decades, rice yield was not considerably improved and appeared to reach a plateau. Molecular breeding is widely accepted as an effective solution to improve the breeding efficiency. A few efforts have been made for yield improvement via gene pyramiding and rational design^1–3^, however, such efforts depends largely on precise genetic dissection of agronomic traits due to the fact that rice yield is a complex trait controlled by both major-effect and minor-effect genetic loci^3^. Though a large number of reports on QTL mapping and cloning of yield-related loci have been available^4, 5^, information about the QTLs or alleles in many elite parental lines is still lacking, which might prevent efficient use of them in molecular breeding^6^. In addition, yield related traits such as panicles per plant, grains per panicle, and thousand-grain weight could influence each other. The interaction networks underlying the complex traits of yield are still largely unknown.

Due to the rapid development of next generation sequencing technology, genotyping by sequencing (GBS) has gained tremendous popularity as a rapid and cost-effective method in the development of genome-wide markers for genetic studies^7^. High-throughput SNP genotyping has been used in a number of QTL mapping studies in rice^8–10^. For instance, Duan *et al*.^9^ used MSG sequencing in the QTL mapping of a giant panicle rice accession R1128 and detected 49 QTLs for five yield traits^9^. Chen *et al*. performed high-resolution QTL mapping for grain appearance traits in *indica* rice^10^. As a reduced-representation sequencing approach of multiplexed samples, restriction-site associated DNA sequencing (RAD-seq) is a useful and cost-effective tool for genetic mapping studies^11^. RAD-seq approach focuses only on short fragments of DNA adjacent to a particular restriction enzyme in the genome and provides significant data complexity reduction and increased throughput, thus allowing efficient and high density SNP discovery and genotyping.

To better understand the genetic basis of rice yield traits in an elite restorer, we constructed a set of recombinant inbred lines (RILs) population derived from the cross between *japonica* inbred Francis and *indica* restorer R998. The two parents have similar thousand grain weight but different tillering capacity and panicle type. Francis has low tiller numbers and large panicles, while R998 has high tiller numbers and medium-sized panicles. High density bin map was generated after low-coverage RAD-seq of each RIL line and used for QTL mapping of 6 yield related traits including thousand grain weight (TGW), panicles per plant (PPP), grains per panicle (GPP), above-ground biomass (AGB), grain yield per plant (GYP) and harvest index (HI). Due to the fact that R998 is an elite restorer line widely used in commercial hybrid rice production, with 16 hybrid combinations released in the market, genetic dissection of yield formation in such an elite line is useful for breeders and may provide insightful guidance in high-yielding molecular breeding.

## Results

### Sequence analysis and construction of bin-map

**Figure 1:**
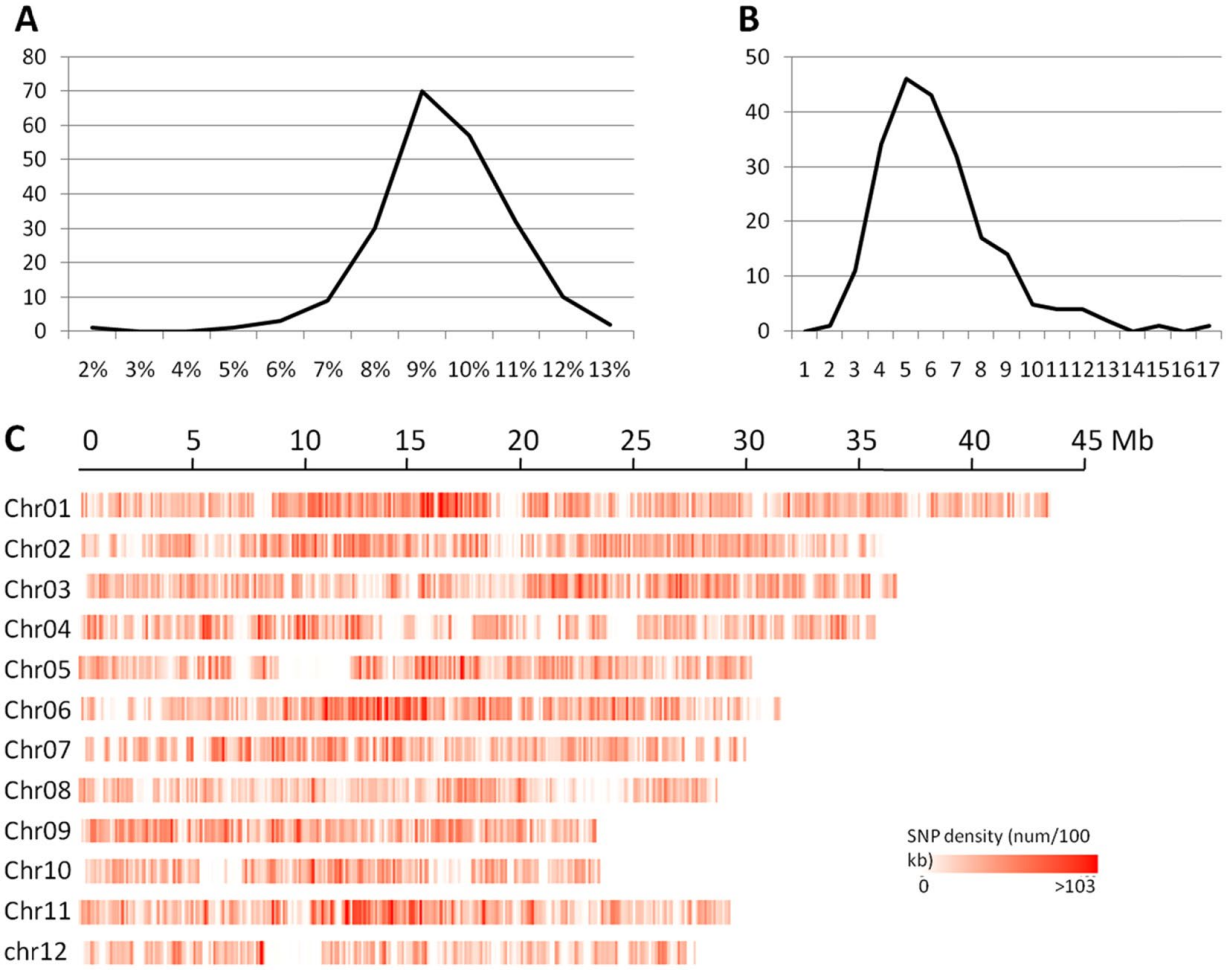
Sequencing results for the RILs population. (**A**) Coverage distribution in the RILs population. The horizontal axis represents coverage and the vertical axis indicates the number of individual lines. (**B**) Distribution of sequencing depth in the RILs population. The horizontal axis represents the sequencing depth and the vertical axis indicates the number of individual lines. (**C**) Heat map distribution of SNPs on each chromosome of the rice genome.

Two parents and a total of 213 RILs were used for RAD-seq and resulted in a total amount of 46.93 Gb raw data, with an average of 218.28 Mb for each individual, among which the raw data for 197 individuals were over 100 Mb. Following the procedure of sliding-widow method^12^, we used a window size of 15 SNPs. One SNP was sliding each time and the genotype of that window was obtained. Thus we got the genotypes of each individual and eventually obtained a total of 3016 bin markers. The length of bin markers ranged from 20 kb to 4.4 Mb (Supplementary Figure S1), with a mean of 123.8 Kb, indicating the presence of recombination breakpoint every 123.8 kb in average. In total, 87.4% of bin markers were less than 0.25 Mb in length. There were 83 bins larger than 0.5 Mb in size and 4 large bins over 3.0 Mb dispersed on chromosomes 3(chr03_bin178), 4(chr04_bin57), 8(chr08_bin87) and 11 (chr11_bin98). The number of SNPs and bins per chromosome is shown in Table 1. Further, we generated a high-density genetic linkage map with a total distance of 3646.19 cM using the 3016 bin markers (Fig. 1, Supplementary Figure S2). The average distance between two bin markers across the map was 1.21 cM. The number of bin markers on different chromosomes ranged from 174 on chromosome 12 to 389 on chromosome 1 (Table 1). For each chromosome, the average genetic distance between adjacent bins ranged from 0.76 to 1.84 cM, with the maximal distance between 3.44 and 10.61 cM.

**Table 1:** Number of SNPs and bins per chromosome in the R998 × Francis RILs population.

Chromosome	Number of SNPs in population	Number of bins	Length of genetic distance (cM)
Chr01	13747	389	296.371
Chr02	10533	337	396.433
Chr03	10831	334	227.044
Chr04	8332	278	399.559
Chr05	7650	246	332.199
Chr06	9955	234	269.438
Chr07	7994	228	377.276
Chr08	6062	214	249.178
Chr09	7766	195	220.843
Chr10	5543	179	329.698
Chr11	8924	208	272.52
Chr12	4565	174	275.632
Total	101902	3016	3646.191

### Quality and accuracy of the map

To examine the quality of the constructed bin-map, we first compared the linkage map with a map generated from an RIL population of 150 individuals derived from a cross between *indica* cv. 93–11 and *japonica* cv. Nipponbare^8^. The total number of bin markers in Wang’s map is 2,334, and that of our map is 3016. The total genetic distance of the 12 chromosomes of these two maps is quite divergent, with 1539.5 cM in their map and 3646.19 cM in our map. It appeared that the total genetic distance of our map is almost twice bigger. As a result, the average genetic distance between adjacent bins with greater than zero distance is 1.21 cM on our map, larger than the average of 0.72 cM in Wang’s map. The maximal genetic distance between adjacent markers is similar, with 10.61 and 8.3 cM on our map and Wang’s map, respectively. In general, the marker distance appeared to be larger than Wang’s map, indicating the algorithm difference between the software MSTMap we used and MAPMAKER for construction of the genetic map.

**Figure 2:**
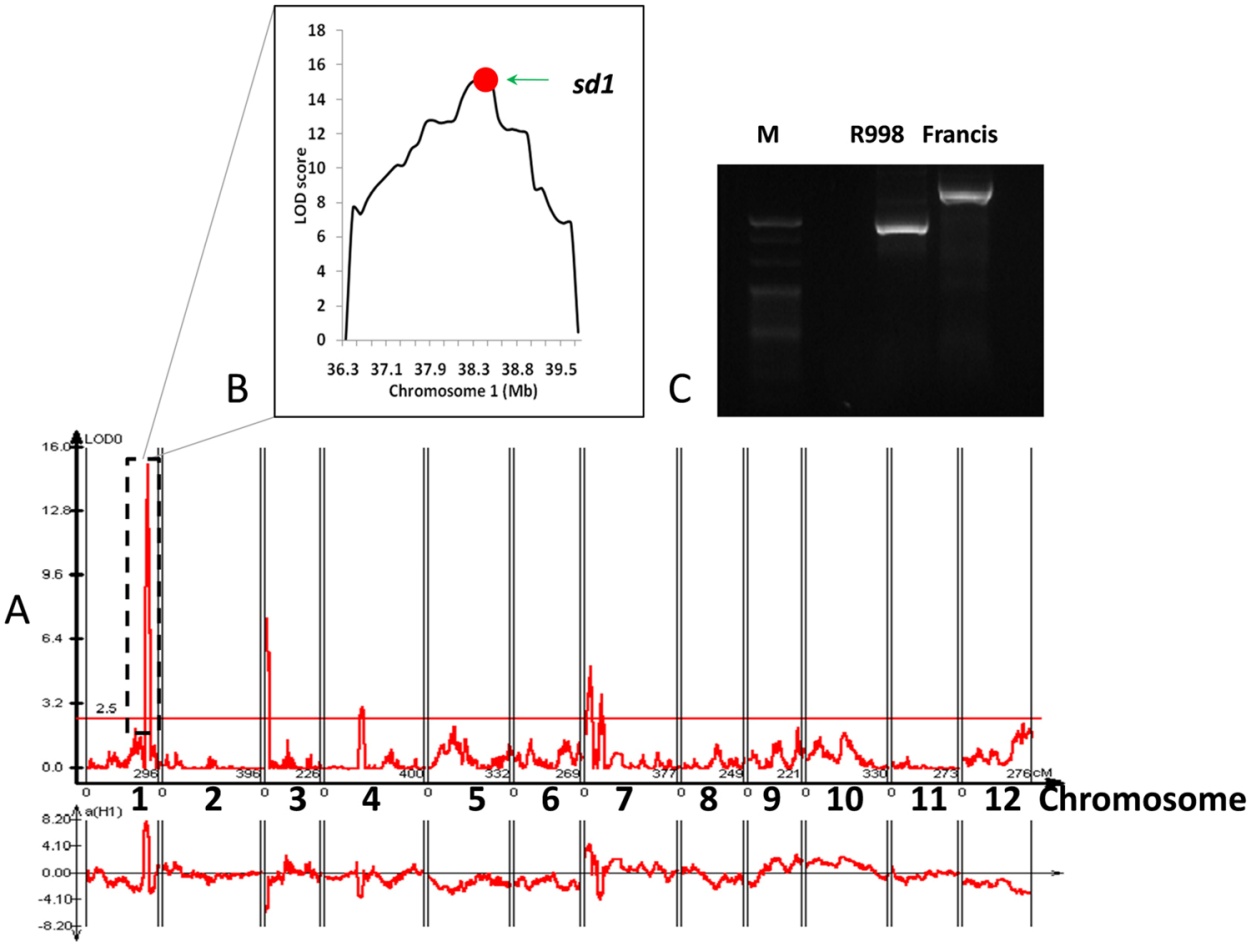
Mapping of QTLs controlling plant height in RILs population and the location of *sd1*. (**A**) Mapping curve of QTLs controlling plant height on 12 chromosomes.Curves in plot indicate the genetic or physical coordinate (X-axis) and LOD score (Y-axis) of detected QTL. (**B**) The box inside is the zoom-in image of the peak on chromosome 1. Red dot presents the relative physical position of *sd1* gene. (**C**) Genotyping of the two parent lines using a functional marker of *sd1*. M, 20 bp ladder (TaKaRa).

The accuracy and mapping resolution of the map was examined from mapping of *sd1*, the well-known semidwarfing gene, which is also known as the “green revolution gene”^13^. We scored the plant height for the 213 RILs lines and mapped the phenotype with CIM method in WinCartographer 2.5. As a result, seven QTLs associated with plant height were detected. Among them, the peak of *qPH1.2* overlapped with the cloned gene *sd1* at chr01_bin331 with a high LOD value of 15.18. The physical distance between *sd1* gene and chr01_bin331 is only 0.6 Kb, demonstrating a high mapping resolution of the bin-map (Fig. 2A and B). In parallel, we verified the presence of a null allele of *sd1* in R998 and a wild type allele of *Sd1* in Francis using a gel-based functional marker described previously^14^ (Fig. 2C).

### Distribution of yield traits and trait correlations

**Figure 3:**
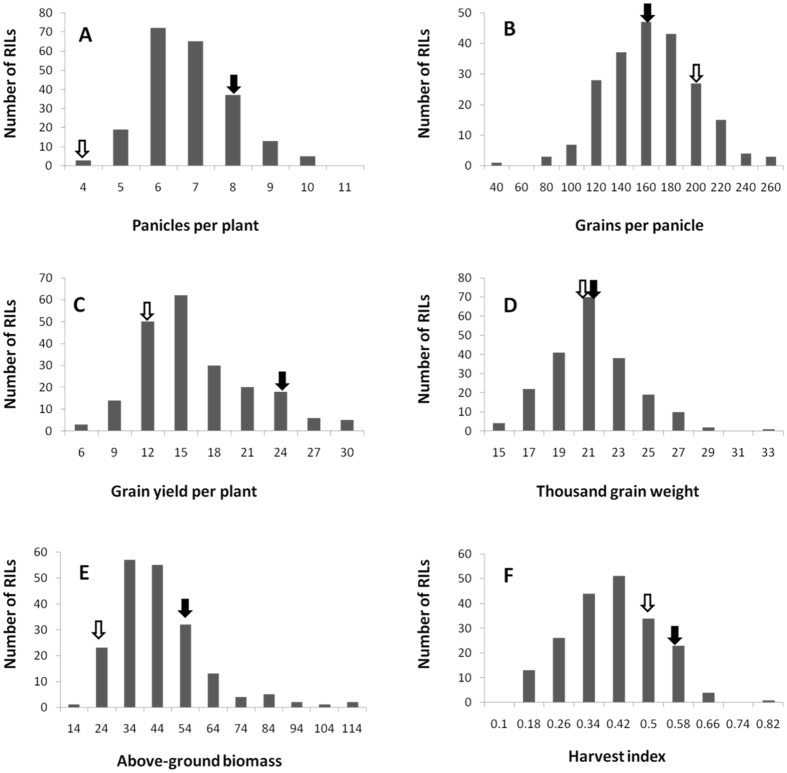
Frequency distribution of phenotypes for yield traits in the RILs population. (**A**) Frequency distribution of panicles per plant. (**B**) Frequency distribution of grains per panicle. (**C**) Frequency distribution of grain yield per plant. (**D**) Frequency distribution of thousand grain weight. (**E**) Frequency distribution of above-ground biomass. (**F**) Frequency distribution of harvest index. Black arrows represent trait means of R998, while white arrows indicate trait means of Francis.

There are considerable variance between two parental lines regarding the panicles per plant, grains per panicle and yield per plant (Table 2). However, there is only slight difference in thousand-grain weight between two parents. Taken together, Francis is a variety with low tillering capacity, big panicle and relatively low yield, whereas R998 is featured by high tillering capacity, medium-sized panicles and high yield. All the six traits displayed a normal distribution within the RILs population. Most of them showed transgressive segregation except the number of panicles per plant (Fig. 3). Both the degree of skewness and kurtosis were mostly less than 1, indicating their nature as quantitative traits controlled by multiple genes and thus meeting the requirements of QTL analysis.

**Table 2:** Means of yield traits for the parental lines and the RILs population.

Traits	Parent		RILs Population			
	Francis	R998	Mean ± SD	Range	Kurtosis	Skewness
PPP	3.0	8.0	6.4 ± 1.14	3.4–10.0	0.52	0.54
GPP	190.0	155.1	153.0 ± 35.6	35.4–255.1	−0.01	0.49
GY	10.4	23.2	14.8 ± 5.0	3.0–31.4	0.70	0.02
TGW	20.96	21.23	20.31 ± 2.80	14.28–31.87	0.88	0.60
AGB	20.6	45	40.05 ± 16.11	13.8–108	3.35	1.50
HI	0.50	0.52	0.37 ± 0.12	0.10–0.79	−0.07	0.15

The correlation between yield traits was evaluated by regressing phenotypic values of one trait on those of another. The correlation coefficients among traits are shown in Table 3. Most of the correlations were in the positive direction, consistent with previous studies^15^. For instance, grain yield per plant is positively correlated with panicles per plant, grains per panicle, thousand grain weight, above ground biomass and harvest index. Three pairs of traits have negative correlations, such as panicles per plant and grains per panicle, thousand grain weight and grains per panicle, and above ground biomass and harvest index.

**Table 3:** Correlation coefficients (r) among yield traits in the RILs population derived from the cross of R998 × Francis.

trait	PPP	DPP	GY	TGW	AGB	HI
panicles per plant (PPP)	1					
grains per panicle (GPP)	−0.137	1				
grain yield per plant (GY)	0.469**	0.366**	1			
thousand grain weight (TGW)	0.087	−0.134	0.351**	1		
above-ground biomass (AGB)	0.315**	0.215**	0.544**	0.194**	1	
harvest index (HI)	0.149*	0.272**	0.574**	0.227**	−0.281**	1

Note: * and ** indicate significant difference at the 0.05 and 0.01 level, respectively.

### Detection of yield-related QTLs

**Figure 4:**
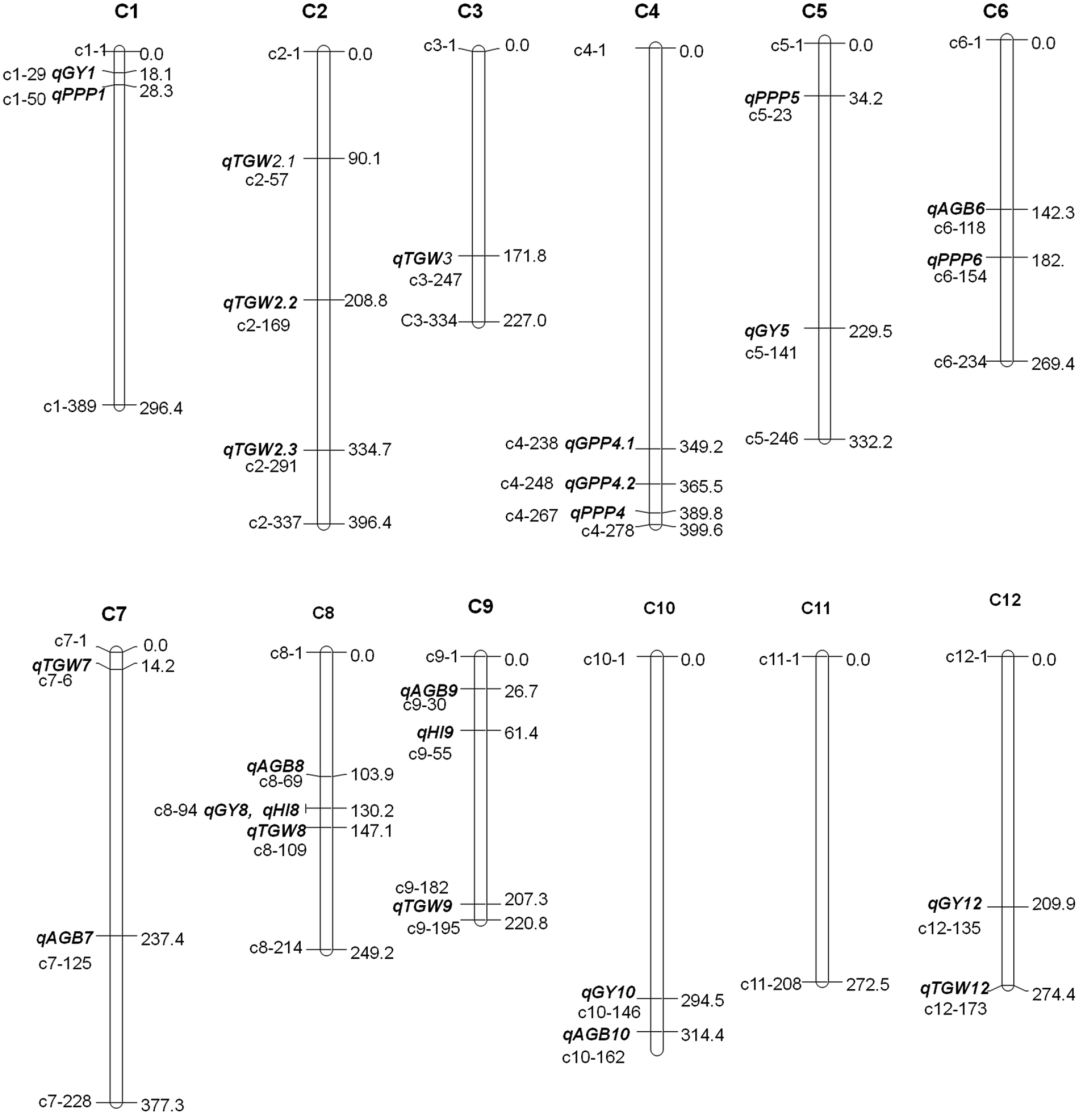
QTL locations of yield-related traits on the bin map. Bin numbers and genetic distance (cM) from the distal end of the short arm of each chromosome are shown. Detected QTLs were marked in bold italic letters. PPP, panicles per plant; GPP, grains per panicle; GY, grain yield per plant; TGW, thousand grain weight; AGB, above-ground biomass; HI, harvest index; C, chromosome. Bin markers were displayed in short form, for instance, c1-1 refers to bin 1 of chromosome 1.

We used 213 RILs derived from the R998 × Francis cross for mapping QTLs involved in yield formation. Using the composite interval mapping (CIM) in Windows QTL Cartographer 2.5, we detected a total of 26 yield-related QTLs in the population. Significant QTLs were detected for all the six traits as summarized in Table 4 and Fig. 4.

**Table 4:** QTLs for yield components identified in the R998 × Francis RILs population.

QTL	Chr	LOD value	peak marker	genetic position (cM)	physical position(bp*)	marker length(bp)	additive	Percentage of variance explained(%)
*qPPP1*	1	4.45	chr01_bin50	28.3	3857577–3925287	67711	−0.37	7.3
*qPPP4*	4	5.65	chr04_bin267	389.8	34229022–34276617	47596	−0.41	9.07
*qPPP5*	5	3.13	chr05_bin23	34.2	2073261–2110577	37317	−0.31	5.54
*qPPP6*	6	2.72	chr06_bin154	182.4	23728747–23828415	99669	−0.28	4.63
*qGPP4.1*	4	2.51	chr04_bin238	349.2	31749920–31774872	24953	9.98	6.9
*qGPP4.2*	4	2.72	chr04_bin248	365.5	32374932–32588914	213983	9.98	7.47
*qGY1*	1	3.94	chr01_bin29	18.11	2119506–2215651	96146	−1.44	5.45
*qGY5*	5	3.59	chr05_bin141	229.51	19881099–19940641	59543	−1.50	6.37
*qGY8*	8	13.46	chr08_bin94	130.21	16465630–16590676	125047	−2.70	21.22
*qGY10*	10	4.39	chr10_bin146	294.51	20352219–20619333	267115	−1.51	6.84
*qGY12*	12	4.33	chr12_bin135	209.91	23675639–23803476	127838	−1.54	6.81
*qTGW2.1*	2	2.56	chr02_bin57	90.11	5657047–5869449	212403	−0.12	4.05
*qTGW2.2*	2	3.55	chr02_bin169	208.81	22277253–22374393	97141	−0.16	4.97
*qTGW2.3*	2	3.08	chr02_bin291	334.71	32504342–32555810	51469	0.16	4.27
*qTGW3*	3	6.7	chr03_bin247	171.81	28494281–28520941	26661	−0.20	11.52
*qTGW7*	7	2.66	chr07_bin6	14.21	1190683–1237988	47306	0.11	3.12
*qTGW8*	8	12.53	chr08_bin109	147.11	18841775–19053506	211732	−0.26	18.64
*qTGW9*	9	3.88	chr09_bin182	207.31	21438495–21495270	56776	−0.14	5.71
*qTGW12*	12	4.97	chr12_bin173	274.41	27146979–27257344	110366	0.17	8.56
*qAGB6*	6	2.7	chr06_bin118	142.3	19988889–20195160	206272	4.02	6.09
*qAGB7*	7	2.54	chr07_bin125	237.4	19443803–19498463	54661	−3.99	5.87
*qAGB8*	8	3.36	chr08_bin69	103.9	5855674–5897395	41722	−5.11	8.51
*qAGB9*	9	2.72	chr09_bin30	26.7	6793297–6873993	80697	9.00	11.66
*qAGB10*	10	3.16	chr10_bin162	314.4	21818948–21843781	24834	−4.15	6.19
*qHI8*	8	9.31	chr08_bin94	130.2	16465630–16590676	125047	−0.05	19.92
*qHI9*	9	2.95	chr09_bin55	61.4	9269614–9407427	137814	−0.03	6.89

Note:“*” denote physical positions based on the MSU Rice Genome Annotation Project release 7 (http://rice.plantbiology.msu.edu/). PPP, panicles per plant; GPP, grains per panicle; GY, grain yield per plant; TGW, thousand grain weight; AGB, above-ground biomass; HI, harvest index.

Of the QTLs for yield components, four QTLs were associated with panicles per plant. R998 alleles contributed positively to all the four loci. They were located on the chromosomes 1, 4, 5, and 6 respectively, thus terming *qPPP1*, *qPPP4*, *qPPP5* and *qPPP6*. The phenotypic variation explained by the individual QTLs ranged from 4.63% to 9.07%.

Two QTLs (*qGPP4.1* and *qGPP4.2*) were detected for grains per panicle on chromosome 4, and the positive alleles were attributed to Francis. They explained a phenotypic variation of 6.9% and 7.47%, respectively.

Five QTLs (*qGY1*, *qGY5*, *qGY8*, *qGY10* and *qGY12*) associated with grain yield per plant were detected on chromosome 1, 5, 8, 10 and 12, respectively and the explained phenotypic variance ranged from 5.45% to 21.22%. The R998 allele contributes to the increase of grain yield per plant at all the five loci. One of the QTLs, *qGY8* showed the largest effect and explained 21.22% of the phenotypic variance.

Eight QTLs were detected for thousand grain weight, explaining 4.05% to 18.64% of the phenotypic variance in the population. At three of the loci, the Francis alleles conferred a positive effect in increasing the thousand grain weight. While the R998 alleles at the other five QTLs were contributing positively to thousand grain weight. Particularly, *qTGW3* and *qTGW8* are the two QTLs with the largest additive effect on thousand grain weight. And R998 alleles contribute positively at both loci and accounted for 11.52% and 18.64% of the variance for thousand grain weight in the population.

Five QTLs were associated with above ground biomass, locating on chromosome 6, 7, 8, 9 and 10, respectively. They explained a phenotypic variance ranging from 5.87% to 11.66%. Among them, Francis allele conferred a positive effect at *qAGB6* and *qAGB9*. While for the other three loci *qAGB7*, *qAGB8* and *qAGB10*, R998 alleles contributed positively for the formation of above ground biomass.

**Figure 5:**
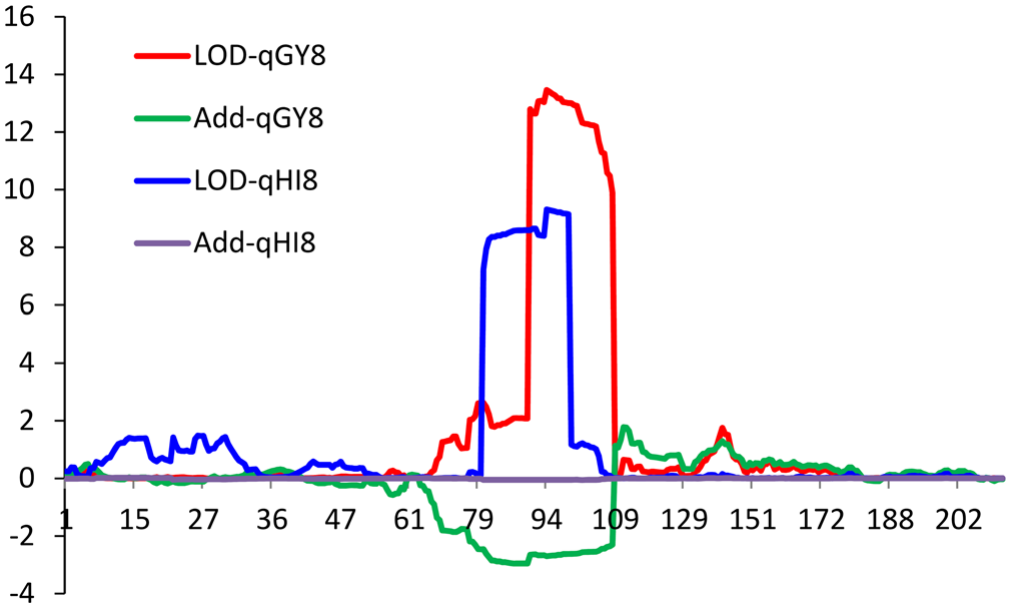
Co-localization of *qGY8* and *qHI8*.The value of LOD and corresponding additive effect for the trait of grain yield per plant (GY) and harvest index (HI) on chromosome 8 were shown. Y axis indicates LOD value or additive effect. X axis indicates the bin numbers on chromosome 8.

Two QTLs were detected for harvest index. Both alleles of *qHI8* and *qHI9* from R998 are responsible for increasing the harvest index, explaining a phenotypic variance of 19.92% and 6.89%, respectively. Notably, *qHI8* and *qGY8* coincided at the same location, with a LOD peak at chr08_bin94 (Fig. 5).

### Validation of *qGY8* and molecular marker development

To validate the QTLs for grain yield, we repeated the phenotyping of grain yield per plant in 2015 and ran the QTL detection for a second time. As a result, four out of the 5 QTLs associated with grain yield per plant was repeatedly detected, locating on chromosome 1, 5, 8, and 12 respectively. They explained a phenotypic variance of 5.02%, 7.39%, 34.97% and 6.85%, respectively. The QTL locations are exactly the same as those detected in the year 2014. Especially for the major locus *qGY8*, it explained a phenotypic variance of over 20% in both years.

**Figure 6:**
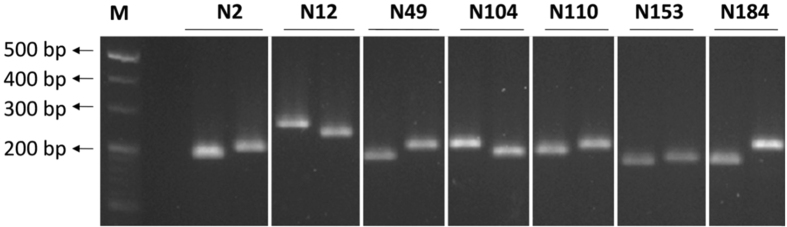
Development of tightly linked InDel markers in the *qGY8* region. M, 20 bp DNA ladder (TaKaRa). For each pair of samples, the left well represents R998 and the right well stands for Francis, respectively. PCR products were separated on 3% agarose gel. The full-length gel is presented in Supplementary Figure 3.

Further, we performed genome re-sequencing of both Francis and R998 in 32x depth. The sequencing data provides useful information to compare genomic variation in the QTL region between the parental lines. Moreover, we developed seven InDel markers within the peak region of *qGY8* according to the polymorphism between the parents (Supplementary Table S1). All of the newly developed markers showed the significant polymorphism between Francis and R998 (Fig. 6), which will be used in marker assisted selection (MAS) and fine mapping of *qGY8* locus.

## Discussion

It is widely accepted that mapping population derived from parents divergent in genetic composition and/or phenotype could yield better QTL mapping results^16^. We used a recombinant inbred line (RILs) population derived from Francis and R998, which belong to *japonica* and *indica* subspecies respectively and are diversified in several agronomic traits such as tiller number, grain yield and plant architecture. Therefore, as revealed from the distribution pattern of phenotype and polymorphic SNP sites, our RILs population is ideal for QTL mapping. The male parent R998 is an elite *indica* restorer line widely used in three-line hybrid rice seed production in China. It has high combining ability, strong restorability and broad restoring spectrum^17^. According to the statistics of ricedata website (www.ricedata.cn), R998 is listed as one of the most-widely used restorer lines during the year of 1983–2010, together with the famous restorer Minghui 63. The R998-series hybrid rice combinations such as Tianyou 998, Boyou 998, Qiuyou 998 and Shanyou 998 were widely grown in south China, with an accumulated growing area reaching over 4.5 million hectares up to today. Therefore, QTL mapping of yield traits using such elite line would provide useful insight into the genetic basis of yield formation, as well as into further exploration of its breeding value in the context of rational super rice design through the approach of gene pyramiding.

A total of 101,902 high quality SNPs were identified in the genome between the *indica* line R998 and *japonica* line Francis using low-coverage RAD-seq approach. As a result, a high-density genetic map containing 3016 bin markers was generated, which had higher resolution than traditional gel-based SSR markers or RICE6K SNP^18^. As revealed in a previous study by Yu *et al*.^7^, increased density marker will considerably improve the QTL mapping resolution and the mapping accuracy. The high-density genetic map constructed in this study is an ideal map for QTL mapping or map-based cloning.

In this study, we detected in total 26 QTLs controlling 6 yield-related traits and located their physical positions on the corresponding chromosomes. Traditionally, lack of shared markers often prevented a precise comparison of QTLs detected between different studies^19^. In our study, however, the available marker information with physical positions in the bin map considerably facilitated the comparison of QTLs with earlier studies. The QTLs were distributed over all the chromosomes except chromosome 11. Among them, four genetic intervals were associated with panicles per plant. *qPPP1* on the short arm of chromosome 1, together with the *qPN1* locus by Tian *et al*.^20^ and AQDY009 locus by Kobayashi *et al*.^21^ are located in the same interval. *qPPP4* was mapped to the same interval as reported previously by multiple studies in different populations^15, 22–24^. Additionally, CQK6 mapped by Liao *et al*.^25^ on the chromosome 5 was in the similar location of *qPPP5* in this study. *qPPP6* on chromosome 6 was co-located with AQDY102^21^ (Gramene QTL accession) and CQJ9^25^.

Francis has an average panicle size of 190 grains per panicle, whereas that of R998 is only 155. Not surprisingly, both QTLs associateda with grain per panicle were contributed by the male parent Francis. Both QTLs *qGPP4.1* and *qGPP4.2*, were found on the end of the long arm of chromosome 4. Previous studies revealed several QTLs at similar position with *qGPP4.1*, such as *gp4* ^26^, *gn-4* ^27^ and AQDR053^28^. Likewise, *qGPP4.2* appeared to be overlapped with the *qSPP4* locus^29^.

Grain yield is one everlasting goal in rice breeding. In our study, five loci associated with grain yield per plant were detected and four of them were consistently detected within two years thus they were stably expressed QTLs. They were distributed on chromosome 1, 5, 8, 10 and 12, respectively. All the positive-effect alleles for the trait of grain yield per plant were contributed by R998 alone, which indicated that the elite line R998 is pyramiding all the superior alleles. The *qGY1* locus we detected is within the same interval as *qGY1* reported by Wang *et al*.^30^ (physical position chr01:1,736,105- 2,289,674 bp, IRGSP 1.0). Close to the position of *qGY1*, three other QTLs controlling grain yield were reported including *yld1.1* ^31^, *GYP1* ^32^ and *yld1.1* ^33^.

In the same location of *qGY5* locus, *qHI-5* ^34^, *yd5a* ^35^ and *qgwt5* ^23^ were reported to be responsible for grain yield per plant. For *qGY10*, no QTL for grain yield per plant was reported at the same location. However, Li *et al*.^28^ described a QTL controlling grain weight per panicle in its vicinity. *qGY12* has a similar location as the locus *AQCN015* (Gramene QTL accession)^36^. Similarly, *yd12* for the trait of grain yield was detected 3.1 Mb away from *qGY12* on chromosome 12^37^. Notably, *qGY8* is a major effect QTL contributing to grain yield per plant and three QTLs for grain yield at the same location were reported, such as GRYLD^28^, *yd8* ^35^ and *yld8.1* ^38^. In two successive years, *qGY8* explained a phenotypic variance of over 20% which most probably was attributed to artificial selection in breeding process. To facilitate further precise selection of *qGY8*, we developed seven tightly linked co-dominant InDel markers according to sequence variation between the two parents at the LOD peak site. These markers could be adopted in marker-assisted selection scheme for gene introgression and gene pyramiding.

Grain weight is an important factor contributing to grain yield. In our study, we detected a total of 8 loci associated with grain weight, among which three were located on chromosome 2. Compared to previous studies, we found QTLs with similar location as *qTGW2.1*. For example, *Gwt1b* ^39^ and *QKw2b* ^28^ were found at the similar site. For *qTGW2.2*, it shared the same location with *QKw2a* ^28^. Two other QTLs linked to grain weight, *gw2.1* ^19^ and *gw2.1* ^31^, were detected at the same position as *qTGW2.3*. On chromosome 3, we detected *qTGW3* at the position of 28,494,281-28,520,941 bp, which is very close to *qGW-3-1* ^40^ (Chr3: 29,052﻿,﻿279 bp) and *gw3.2* ^19^ (Chr3: 33,393,485 bp), but 12 Mb away from the well characterized *GS3* locus (chr03:16,729,501-16,735,109). *qTGW7* (1,190,683-1,237,988 bp) was sharing similar location as *gw7.1* at the end of the short arm of chromosome 7^31^. *qTGW8* (Chr8:18841775–19053506 bp) was overlapping with *qGW8-1* ^41^ and *HGW8* ^32^. In another study by Xie *et al*.^42^, the major QTL *GW8.1* controlling grain weight was fine mapped to an interval of 306.4 kb between the marker RM23201.CNR151 and RM30000.CNR99. A (Chr8: 21,523,421-21,829,999 bp). Though the physical distance between *qTGW8* and *GW8.1* is over 2.5 Mb (ca. 10 cM in genetic distance), we were not able to completely exclude the possibility that *qTGW8* in the present study is identical to *GW8.1*. Further fine mapping of *qTGW8* is required to verify this point. For *qTGW9* with a LOD peak at 21,438,495–21,495,270 bp, it is located nearby *gw9.1* ^19^ and in the same interval of CQAS114^15^. Interestingly, it exactly coincided with *gw9* ^43, 44^, which was flanked by markers RM24718.CNR113-RM30005.CNR142 (Chr9:21,205,446-21,222,285 bp). Considering the small physical distance of 216 kb between *qTGW9* and *gw9*, it is most probably that *qTGW9* is identical to *gw9*. Further, we detected a QTL for grain weight at the position of 27,146,979–27,257,344 bp on the long arm of chromosome 12, which is in the vicinity of a similar locus detected by Hua *et al*.^26^ (Chr12: 23,799,065-24,360,652 bp).

Five QTLs associated with above-ground biomass were detected in this study, locating on chromosome 6, 7, 8, 9 and 10, respectively. *qAGB7* is in the same location as AQGI131^45^. *qAGB8* shared a similar location as AQGI263^45^, which was detected as a QTL for dry weight in the seedling stage. *qAGB9* was in the same location as *qTDW9-1* (AQAC016, 6016329 bp). *qAGB10* is coincided with the QTL loci including AQEX020 (*qSDW10*)^46^, AQGI173^45^ and AQGJ029^47^. However, no QTL for biomass was reported around the QTL *qAGB6*. Therefore, *qAGB6* detected here is considered a novel QTL.

Harvest index is important trait in breeding and it represents the ability in the allocation of carbohydrate in the grains rather than in the vegetative part. We detected two loci associated with harvest index on chromosome 8 and 9, respectively. *qHI8* is almost in the same interval as *qGY8* (Fig. 5). In a previous study by Hittalmani *et al*.^24^, *qHI8-1* and *qHI8-2* (Chr8: 17,437,513-17, 438, 003 bp) were reported at similar location with *qHI8* (LOD peak position 16,465,630-16,590,676 bp). In the interval of *qHI9*, no QTLs were reported at the same position thus *qHI9* is likely to be a novel QTL for harvest index.

QTLs controlling different traits are sometimes detected at the same or neighboring interval thus forming QTL clusters^6, 19^. QTL clusters for different traits might be explained by tight linkage of multiple causal genes, or by pleiotropic effect of a single gene. We detected a QTL cluster on chromosome 8, with QTLs for grain yield per plant, harvest index and thousand-grain weight at the similar position. Especially for grain yield per plant and harvest index, the interval of *qGY8* and *qHI8* were largely overlapped (Fig. 5). There was positive correlation between harvest index and grain yield per plant, as well as between thousand grain weight and grain yield per plant, therefore the clustering of *qGY8*, *qHI8* and *qTGW8* is reasonable. They showed major effect and the beneficial alleles were from the parent R998. Hence this QTL cluster is a useful target for selection in molecular breeding. Notably, though R998 is pyramiding all the five beneficial alleles for grain yield, there are 7 RILs showing transgressive grain yield over R998, which implies a possible gene interaction between Francis alleles and R998 alleles.

## Methods

### Mapping population

A recombinant inbred line (RILs) population derived from the cross between *japonica* line Francis and *indica* line R998 was used in genetic map construction and in the measurement of yield-related traits. Francis is a tropical japonica cultivar with fewer panicles, large panicle size and lower grain yield per plant while R998 is an elite *indica* restorer line with more panicles, moderate panicle size and higher grain yield per plant. Two parental lines have similar grain weight. The RILs population was obtained from continuous single seed decent (SSD) method. In this study, both parental lines and 213 randomly selected RILs lines (F_8_) were used for genotyping and phenotyping.

### Plant cultivation

In the early seasons of 2014 and 2015, 213 RILs lines and two parental lines were grown in Dafeng Experimental Station of Guangdong Academy of Agricultural Sciences in Guangzhou, China. Rice seeds were sown in March and transplanted at the beginning of April. Randomized block design was used for the experiment. Each line contains 4 rows in the block with 7 plants in each row. The plant density was 16.7 cm × 26.7 cm. All plant materials were grown in paddy fields following normal field management practices.

### Evaluation of the yield-related traits

After maturity, 10 plants were harvested from the middle rows of each line for analysis of 6 yield-related traits including panicles per plant (PPP), grains per panicle (GPP), grain yield per plant (GY), thousand- grain weight (TGW), above-ground biomass (AGB) and harvest index (HI). Evaluation methods were similar to those described by Moncada *et al*.^33^ as follows: (1) Panicles per plant was the average number of panicles on the ten plants, counting the panicles with no less than five seeds. (2) Grains per panicle were measured as the average number of filled grains calculated for the ten plants. (3) Grain yield per plant was the average weight per plant. After removing the empty kernels (grains filled with more than 30% were kept for analysis), bulked harvested filled grains were weighted and divided by 10. (4) Thousand-grain weight was the average weight of 1000 filled grains, measured in grams, averaged over three samples taken from bulk harvested grain from the ten plants. (5) Above-ground biomass was the total dry weight of above-ground part, including the grains and vegetative parts above ground, averaged from ten plants. (6) Harvest index was the dry weight of grains divided by the total dry weight of above-ground biomass evaluated from all ten plants. Correlation among the various traits was calculated in Excel 2007 using the trait averages from the experiments. Skewness and kurtosis were calculated to understand the nature of distribution of yield traits in the RILs population^48^.

### DNA isolation, genome re-sequencing and RAD-seq library preparation

Three weeks after transplanting, leaf samples were harvested from the plants. Genomic DNA was extracted from the parents Francis and R998, and the RILs population using CTAB method with minor modifications. Whole genome re-sequencing was carried out for Francis and R998 at 32x depth by Novogene (Beijing, China). Genome-wide SNP development and genotyping for the RIL population were performed by BGI (Shenzhen, China) using RAD-seq approach as described previously^11^. Bar-coded adapters were designed according to the standard Illumina protocol for paired-end read libraries. One microgram of genomic DNA from each sample was digested with 1 μl FastDigest TaqI (Thermo scientific Fermentas) for 10 min at 65 °C in a 30 μl reaction. Unique barcode adapters (10 μmol) were then added to each sample well. The ligation was done with T4 DNA ligase (Enzymatics) at 22 °C for 1 h and heat inactivated at 65 °C for 20 min. For different samples, twenty-four ligation products were pooled in a single tube and 2 μl chloroform was added to inactivate the restriction enzyme. DNA fragments between 400–600 bp were then selected on a 2% agarose gel and purified using a QIAquick Gel Extraction Kit. All the products were amplified with 10 cycles of PCR (Phusion high-fidelity, Finnzymes) in a volume of 50 μl including 25 μl 2x Phusion Master Mix, 1 μl of common primer (10 μM) and 1 μl index primer. The amplified library was purified using a QIAquick PCR Purification Kit, quantified on the Agilent 2100 Bioanalyzer and sequenced on Illumina Hiseq 2000 platform.

### SNP identification

The sequencing short reads of 100 bp in length of each individual were obtained according to the identified taq sequences. They were aligned with the rice reference genome of Nipponbare IRGSP 1.0 (http://rapdb.dna.affrc.go.jp/) using SOAPaligner program^49^. Consensus sequences for each individual were generated by SOAPsnp. Input file was prepared by SAMtools for SNP calling with realSFS. Population SNP calling was performed with realSFS, based on the Bayesian estimation of site frequency at each site. The likelihoods of genotypes for each individual were integrated and sites with a probability of over 0.95 and a population whole depth higher than 40 were accepted as candidate SNPs. Potential SNPs were then filtered using the following criteria: loci with >70% missing data that also showed serious distorted segregation of the two parental genotypes were excluded. All the SNPs were filtered using a PERL script.

### Determination of recombinant breakpoints

We converted the SNP data into another format to simplify the genotype calling analysis. The SNP type from Francis was coded as “a”, the R998 alleles were coded as “b”, and the heterozygotes were coded as “h”, while missing data was coded as “-”. A sliding window approach proposed by Huang *et al*.^12^ with minor modification was adopted to evaluate a group of consecutive SNPs for genotyping. For each sample, we chose a window size of 15 SNPs without missing data for genotyping calling as described by Duan *et al*.^9^, which covered on average 54 kb or 0.2 cM of rice chromosomes. An a/b ratio of 12:3 or higher was recognized as “a”, while the ratio of 3:12 or lower as “b” and anything in between as “h”. We determined the breakpoints according to a published method for high-throughput genotyping by NGS (next-generation sequencing) with some modifications^9, 50^. Recombination breakpoints were determined by the junction of two different genotypes.

### Construction of Bin map and QTL analysis

Bin genotype was obtained for each individual and used for construction of genetic map. Based on filtering of two parents SNP, a total of 101902 polymorphic SNP was obtained on the whole genome one SNP per 3.6 kb on average. Bin map was constructed using MSTMap software^51^. Data of the genetic map was imported into MapChart 2.2 software and integrated as linkage map^52^. Genetic distances were recorded for each chromosome. For QTL mapping, composite interval mapping (CIM) in Windows QTL Cartographer 2.5 was used for detection of QTL in the whole genome scale, additive effect (a) and phenotypic variance explained (R^2^) was analyzed^53^. The significance threshold of LOD value was calculated using 1000 permutation test. On the significance level of 5%, the LOD value threshold was used as the presence of a QTL. Nomenclature for QTLs followed the guidelines described by McCouch *et al*.^54^.

## Electronic supplementary material

Supplementary information
