# Supplementary material for: QTL mapping using an ultra-high-density SNP map reveals a major locus for grain yield in an elite rice restorer R998
Source: Sci Rep. 2017 Sep 7;7:10914. doi: 10.1038/s41598-017-10666-7 (PMC5589899; doi:10.1038/s41598-017-10666-7)
Supplement: Supplementary file 1 — Supplementary information [file 41598_2017_10666_MOESM1_ESM.pdf]

QTL mapping using an ultra-high-density SNP map reveals a major locus for grain yield in an elite rice restorer R998

Manshan Zhu[1,2+] Dilin Liu[1,2+] Wuge Liu [1,2] Dan Li[1,2] Yilong Liao[1,2] Jinhua Li [1,2] Chongyun Fu[1,2] Fuhong Fu[1,2] Huijun Huang[1,2] Xueqin Zeng [1,2] Xiaozhi Ma[1,2] Feng Wang [1,2] *

Table S1. List of InDel markers developed for *qGY8*

Primer	Forward	Reverse	Genotype score (bp, R998 vs Francis)	Indel position
N2	CTGGCTTACCCTCAAAGCAT	AGCTGGAGACGATTTGCTTG	−24	16467312
N12	TCCCCCAACAATTGATTCTC	TTTTGATTGTTGATTCGAGCTT	22	16471541
N49	ACCCTACGGTACACCCCTCT	CAACCCAGCTAGGGCTTACA	−44	16491993
N104	TTCATTATCCCGCGCATTAC	GAGTGGGAAGCGACATCGT	36	16533954
N110	CCGAAGTGATGATGGGAGAG	CCTCCGTTTCCCTACTTCAA	−30	16542634
N153	GGCGCCATAAATATAGCTACACC	AACGGTGGAGGATGAAAGGT	−20	16559094
N184	GATGTTGTCCGCAGAGATGA	CTCCTCGACAGCATCCACTC	−66	16587529

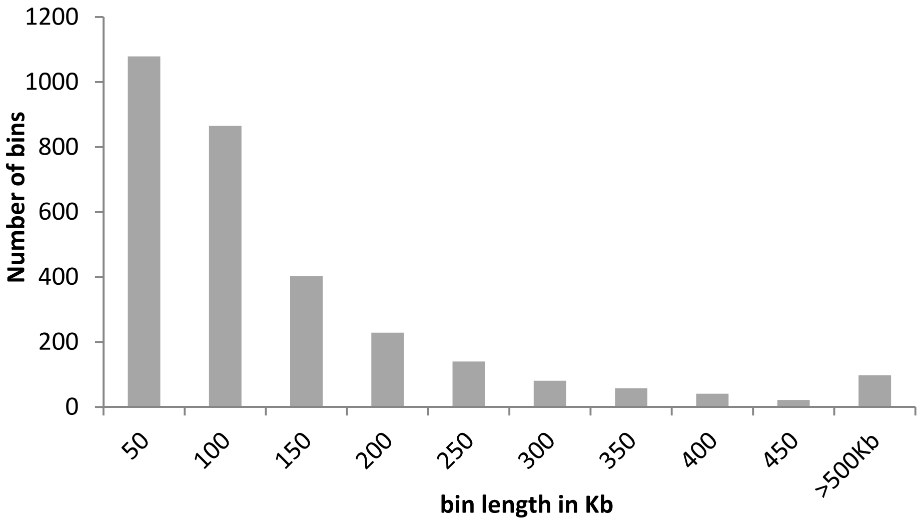

Figure S1. Frequency distribution of bin marker length.

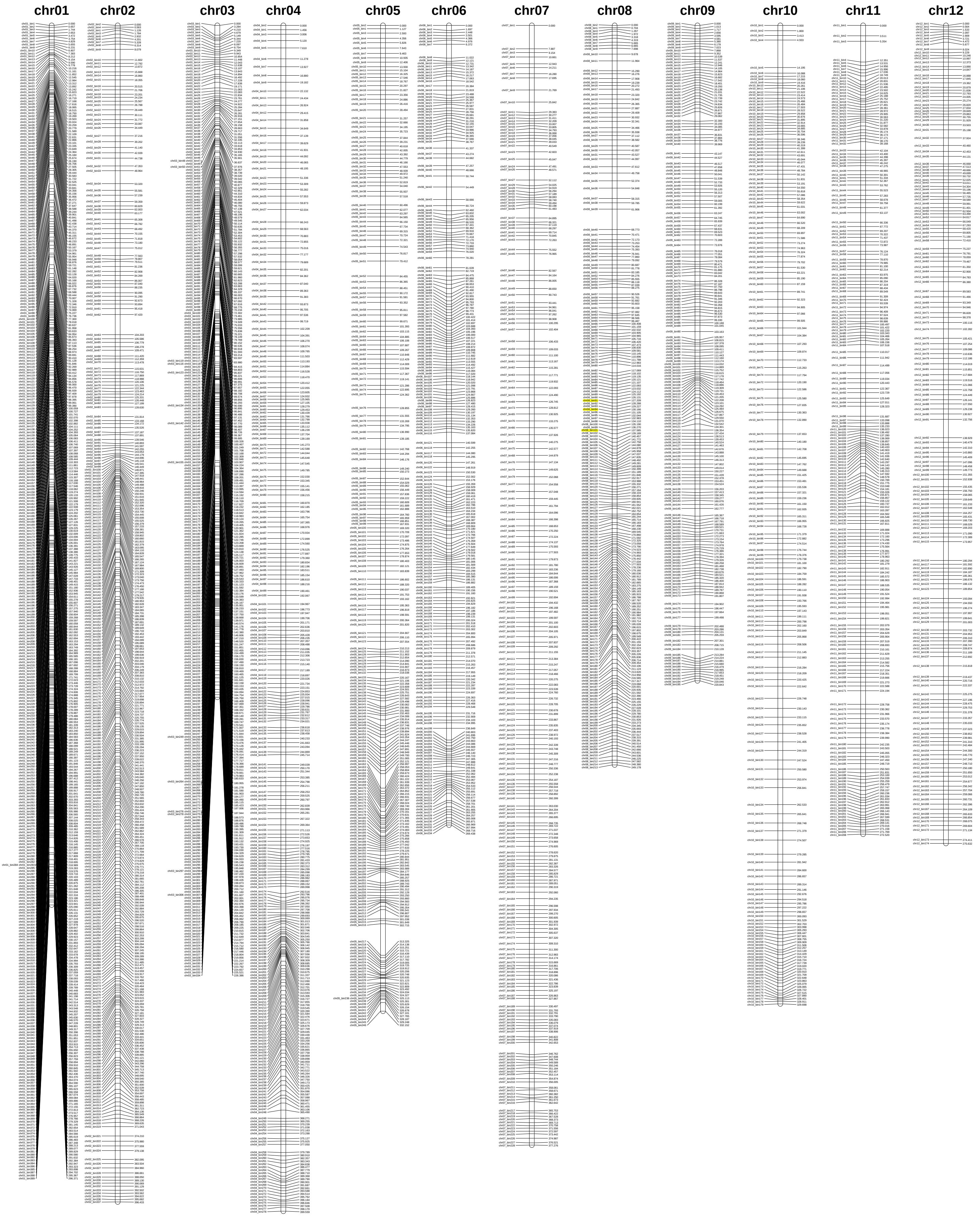

Figure S2. Genetic map constructed from the RILs population of R998 x Francis.

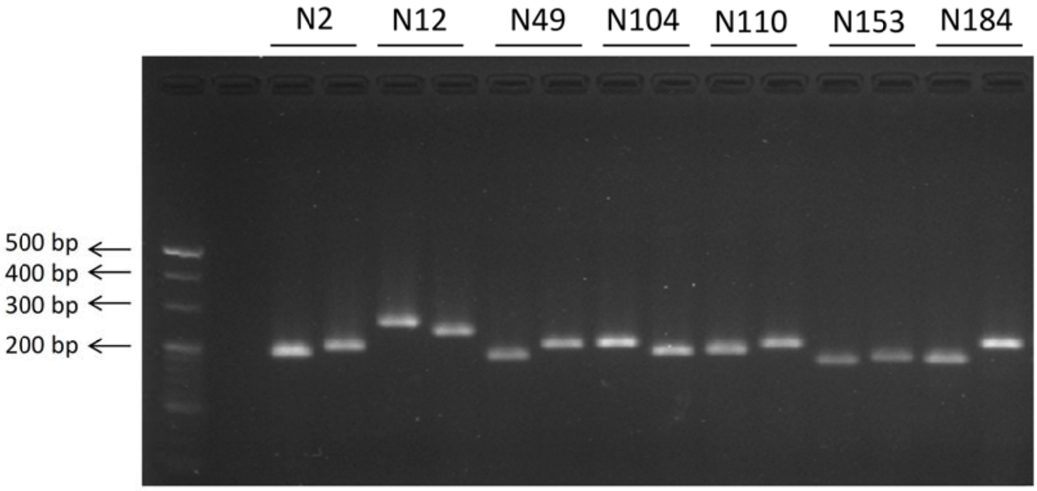

Figure S3. Full-length gel for Fig. 6
